# Global Functional Connectivity at Rest Is Associated with Attention: An Arterial Spin Labeling Study

**DOI:** 10.3390/brainsci13020228

**Published:** 2023-01-30

**Authors:** Shichun Chen, Yakun Zhang, Zongpai Zhang, Tony D. Zhou, Wenna Duan, George Weinschenk, Wen-Ming Luh, Adam K. Anderson, Weiying Dai

**Affiliations:** 1 Department of Computer Science, State University of New York at Binghamton, Binghamton, NY 13902, USA; 2 Department of Psychology, Cornell University, Ithaca, NY 14853, USA; 3 National Institute on Aging, National Institutes of Health, Baltimore, MD 21225, USA

**Keywords:** arterial spin labeling, attention, event-related potential, EEG, P300

## Abstract

Neural markers of attention, including those frequently linked to the event-related potential P3 (P300) or P3b component, vary widely within and across participants. Understanding the neural mechanisms of attention that contribute to the P3 is crucial for better understanding attention-related brain disorders. All ten participants were scanned twice with a resting-state PCASL perfusion MRI and an ERP with a visual oddball task to measure brain resting-state functional connectivity (rsFC) and P3 parameters (P3 amplitudes and P3 latencies). Global rsFC (average rsFC across the entire brain) was associated with both P3 amplitudes (r = 0.57, *p* = 0.011) and P3 onset latencies (r = −0.56, *p* = 0.012). The observed P3 parameters were correlated with predicted P3 amplitude from the global rsFC (amplitude: r = +0.48, *p* = 0.037; latency: r = +0.40, *p* = 0.088) but not correlated with the rsFC over the most significant individual edge. P3 onset latency was primarily related to long-range connections between the prefrontal and parietal/limbic regions, while P3 amplitudes were related to connections between prefrontal and parietal/occipital, between sensorimotor and subcortical, and between limbic/subcortical and parietal/occipital regions. These results demonstrated the power of resting-state PCASL and P3 correlation with brain global functional connectivity.

## 1. Introduction

Attention, which is the ability to maintain focus on a task [1,2,3], plays a pivotal role in many cognitive functions, such as problem solving and reasoning [4]. Attention enables the selection of, focus on, and processing of information [1] and hence involves a wide variety of brain regions [5,6,7,8]. Task-related attentional processes have been frequently linked to various event-related potential (ERP), including the P3 (P300) or P3b component [9,10]. The P3 component is traditionally assessed using an oddball paradigm, in which participants are presented with a sequence of repetitive stimuli interrupted by deviant target stimuli [11]. The amplitude and latency of the P3 component reflect the level of attentional resources and information processing engaged in processing a given stimulus [12] and are therefore widely used in different applications including clinical diagnosis [9,13,14] and cognitive neuroscience [15,16].

P3 amplitude and latency parameters vary widely within and across participants. Variation in these indices have been linked to individual differences in age, gender, sociability, pubertal stages, and genetics [17,18,19,20]. Differences in P3 parameters are also related to individual differences in working memory and fluid intelligence [21,22]. Understanding the neural mechanisms underlying the variation in P3 parameters or attention is crucial for comprehending neuropsychiatric disorders involving attentional impairments (e.g., ADHD [23,24] and bipolar disorder [25,26]).

The P3 has been associated with the activities and morphologies of multiple brain regions: subcortical (e.g., striatum [27], thalamus, insula [28]), prefrontal [28,29,30], and parietal regions [28,29]. Studies have associated P3 components with large-scale networks: the dorsal attention, ventral attention, visual, sensory-motor, and default mode networks [31,32]. The P3 involves information processing among widespread brain regions/networks and so interaction across the whole brain can contribute to P3 characteristics [33].

Interactions across brain regions are frequently described in terms of functional connectivity either during task performance or at rest. Functional connectivity at rest reflects the degree to which the brain can efficiently process task-related information [34,35]. The brain efficiency of functional connectivity at rest measured with resting-state EEG has been found to correlate with P3 amplitude [36] and task performance [37]. Recently, a simultaneous fMRI-EEG study confirmed that resting-state functional connectivity (rsFC) within task-activated brain regions, which is measured from blood oxygenation level-dependent (BOLD) fMRI, is associated with P3 amplitudes [38]. However, the study did not investigate the correlation of whole-brain functional connectivity with P3 parameters. In addition, BOLD fMRI signals are considerably influenced by motion-related (e.g., head movements) or physiology-related (e.g., cardiac pulsations) artifacts [39,40], which may obscure important relations.

In contrast to BOLD fMRI, resting-state dynamic arterial spin labeling (rsDASL) perfusion fMRI is capable of measuring rsFC with less susceptibility to motion artifacts and physiological noises [41]. The reason that rsDASL is less impacted by motion-related noises is that these noises are proportional to the tissue signals and tissue signals are suppressed heavily using the background suppression technique [41]. Given this, rsDASL may hold great potential in robustly exploring the relationship between whole-brain rsFC and P3 parameters, including amplitude and latency. Here, we investigated the role of rsFC measures averaged over the entire brain or global efficiency, measured using rsDASL and resting-state EEG, on the P3. Resting-state EEG was measured for the purpose of comparisons with rsDASL. We hypothesize that rsDASL can provide improved sensitivity in detecting associations between indices of brain global efficiency with P3 parameters and the spatial distribution of P3-involved regions, compared to resting-state EEG. These rsDASL results can not only deepen our understanding of neural mechanisms of P3, but also offer a single global index of neural processes underlying components of attention indexed by the well-known P3.

## 2. Method

### 2.1. Participants

Ten healthy college students (19.20 ± 0.28 years old, age range: 19 to 20 years old, 4 females, 3 left-handed) participated in the study, which served as a convenience sample and was originally designed to study the effect of meditation [42,43]. These participants were recruited from a Binghamton University Meditation course. Per college records, no participants self-identified as having intellectual disabilities. The MRI and EEG/ERP data collection was performed twice in each participant before and after a two-month meditation training course. Participants were compensated USD 50 for each data collection. The Institutional Review Board approved the study, and all participants gave written informed consent. All methods described were carried out following the approved guidelines. We used the data retrospectively to investigate the brain’s global efficiency and its performance in an attentional task. Significant changes in functional connectivity using the MRI data have been reported after meditation training in the same subjects previously [42,43]. In addition, the MRI and EEG/ERP data before and after meditation training were uncorrelated (see details in Appendix A). Therefore, the MRI and EEG/ERP data before and after meditation training were considered to be separate baseline measures in the study, although these data were collected from the same subjects. Alternative baseline analyses were performed with completely independent samples by averaging the global network properties derived from the MRI/EEG data before and after meditation and averaging p3 parameters derived from the ERP data before and after meditation. The results from the alternative baseline analyses confirmed the same trend of association but with reduced statistical power because of a reduced sample size (see Appendix B).

### 2.2. Experiment Settings

#### 2.2.1. MRI Settings

The participants were scanned on a GE 3T MR750 scanner with a 32-channel receive-only phased-array head coil. They were instructed to remain relaxed and keep awake when lying down in the scanner. The scan began with a three-plane localizer to define the anatomy of interest. Sagittal T1-weighted magnetization prepared rapid gradient echo (MPRAGE) images covering the whole brain were acquired in 5 min 30 s with the following parameters: 176 slices with matrix size: 256 × 256; slice thickness: 1.0 mm; echo time (TE): 3.42 ms; repetition time (TR): 7 ms; inversion time (TI): 425 ms; flip angle: 7° field of view (FOV): 25 cm; receiver bandwidth (rBW): 25 kHz. Next, resting-state dynamic pseudo-continuous arterial spin labeling (PCASL) [41] was acquired with a 3D stack of spirals Rapid Acquisition with Refocused Echos (RARE) readout sequence: labeling duration: 2 s; post-labeling delay: 1.8 s; TR: 5 s; field of view (FOV): 24 cm; receiver bandwidth (rBW): 125 kHz. Background suppression was used to minimize the contamination from unstable background tissue signal. For each of the two spiral interleaves, control and label images were acquired consecutively, and therefore, each 3D arterial spin labeling (ASL) volume required 4 TRs (totaling 20 s). Fifty 3D ASL image volumes and a reference volume were collected in 17 min. Perfusion images were used to characterize global network properties because its signals consist of minimal contamination from non-neural-related noises, such as cardiac pulsation, respiratory motion, and participant motion.

#### 2.2.2. EEG and ERP Settings

**Figure 1:**
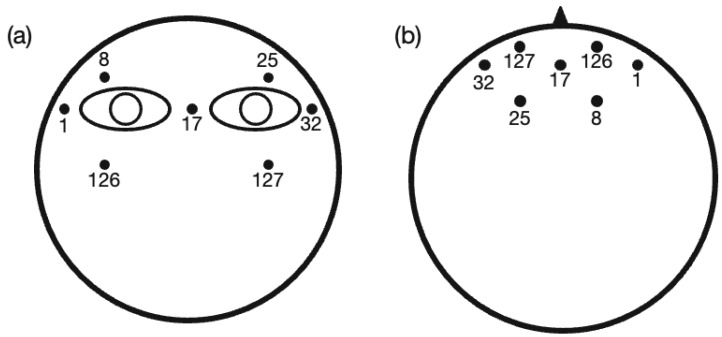
Locations of eye-related electrodes (1,8,17,25,32,126,127) from (**a**) a coronal view and (**b**) an axial view (the triangle standards for the position of the nose) with electrode numbers from the EGI 128-channel Hydro-Cel Geodesic system.

EEG/ERP data were collected outside of the MRI scanner. An EGI 128-channel Hydro-Cel Geodesic system was used for all EEG data collection. The EEG signals were sampled at 250 Hz, and electrode Cz was used as a reference. Vertical electrooculogram (EOG) was recorded from four electrodes (electrodes 8 and 126 are above and below the left eye, electrodes 25 and 127 are above and below the right eye). Horizontal EOG data was recorded from three electrodes (electrodes 32, 1, and 17 are on the left side of the left eye, right side of the right eye, and in the middle of the two eyes, respectively). Illustration of the seven EOG electrodes is shown in Figure 1.

Resting-state EEG data, right after the MRI scan, were recorded during a four-minute eyes-open and four-minute eyes-closed condition. The participants were instructed to sit on a chair and refrain from extensive head motion. Only the eyes-closed resting-state EEG data were used to analyze the global network properties because the eyes-open resting-state EEG data contained more eye blink and horizontal eye movement-related artifacts.

After a 1 min break following the resting-state EEG data collection, a visual oddball task was performed to measure the P3 for each participant. At the beginning of each trial, participants were asked to fixate on the center of a computer monitor. Visual stimuli, “X” and “O”, were randomly presented in the screen center for a duration of 500 ms. The inter-stimulus interval (ISI) ranged from 1.0 s to 2.5 s. During the remaining time (0.5 s to 2 s), the participants were asked to focus on the fixation point. Participants were instructed to press the left button for “X” and the right button for “O.” The EEG experiment consisted of 200 trials in total with 20% of “X” (target, 40 trials) and 80% of “O” (standard, 160 trials).

### 2.3. Data Processing

#### 2.3.1. ASL fMRI Image Processing

The ASL label-control difference image time series was reconstructed using our custom reconstruction algorithm [41,44]. The first 3D ASL difference image was removed to increase stability for further processing. The remaining ASL image time series was realigned to correct for head motion and the mean of the head motion corrected images was generated. For each participant, the realigned ASL image time series was transformed to the standard brain MNI space. Specifically, high-resolution MPRAGE images were segmented into gray matter (GM), white matter, and cerebrospinal fluid. The segmented GM images were co-registered to the mean of realigned ASL images and the co-registered GM images were normalized to a prior GM template in standard MNI space. These normalization parameters were used to transform the realigned ASL image time series for each participant into the standard space.

The ASL regional time series was calculated as mean signal series over the region of interest for each of the 90 regions from the AAL atlas. The coherence of any two regions was used to describe the strength of their functional connectivity, and so a 90 × 90 symmetric coherence matrix was constructed. The coherence value instead of Pearson correlation was used to calculate functional connectivity because coherence in the frequency domain was shown to be more sensitive than the correlation in the time domain [45]. The coherence $C_{xy}\left(f\right)$ represents the interaction of region *x* and *y* at a specific frequency *f* between two signal time series *x*(*t*) and *y*(*t*) and can be expressed as Equation (1):

(1) $$C_{xy}\left(f\right)=\frac{{\left|P_{xy}\left(f\right)\right|}^{2}}{P_{xx}\left(f\right)P_{yy}\left(f\right)}$$

where $P_{xy}\left(f\right)$ is the cross power spectral density function between the two signals *x*(*t*) and *y*(*t*) at frequency *f*, which is the Fourier transform of the cross-correlation function of *x*(*t*) and *y*(*t*). $P_{xx}\left(f\right)$ and $P_{yy}\left(f\right)$ are the auto power spectral density functions at frequency *f*. The coherence value between regions *x* and *y* was estimated by first calling the Matlab function “mscohere” to calculate $C_{xy}\left(f\right)$ with *x*(*t*) and *y*(*t*) as the inputs and then averaging the $C_{xy}\left(f\right)$ across the frequency band [1 Hz, 30 Hz].

#### 2.3.2. Resting-State EEG Data Processing

The resting-state data were preprocessed using the following steps: (1) 1–30 Hz bandpass filter [36] using the EGI Net Station tool; (2) global regression in Matlab to regress the eye blink and horizontal eye movement-related artifacts [46]. Specifically, we used four regressors from EOG electrodes: signals from the upper minus lower electrodes on the left eye (8–126), the upper minus lower on the right eye (25–127), the left horizontal minus middle (32–17), and right horizontal minus middle (1–17); (3) division of the whole data into 10s segments using the EGI Net Station tool; (4) artifact detection using the EGI Net Station tool: segments were marked as bad and rejected if the difference in a moving average of 80 ms between the maximum and minimum voltages is (4a) larger than 200 μV in more than ten channels, or (4b) larger than 55 μV in any 640 ms window.

To facilitate comparison with the broader literature, the 21 canonical electrodes of the 10–20 system were selected to construct the brain network: Fp1 (22), Fp2 (9), F3 (24), F4 (124), C3 (36), C4 (104), P3 (52), P4 (92), O1 (70), O2 (83), F7 (33), F8 (122), T7 (45), T8 (108), P7 (58), P8 (96), Cz (128), Fz (11), Pz (62), Fpz (15), and Oz (75). The numbers in the brackets are the corresponding EGI channels. Because the resting-state EEG data was collected with a reference to Cz, channel 55 close to Cz was used instead.

For each segment of each participant, the coherence for each pair of the 21 channels was calculated (based on Equation (1)) by calling the Matlab function “mscohere” and averaging the coherence values across the frequency band [1 Hz, 30 Hz]. The 21 × 21 coherence matrix was generated for each segment of each participant. The final coherence matrix of each participant was calculated by averaging the coherence matrices across all good segments.

#### 2.3.3. Extraction of P3 Amplitude and Latency from Task EEG Data

The task EEG data was preprocessed using the following steps: (1) 0.5–6 Hz bandpass filter [10] in Matlab; (2) global regression in Matlab to regress the eye artifacts [46] with the same regressors as mentioned in the resting-state EEG eye data for the removal of eye artifacts; (3) data segmentation (100 ms before stimulus onset and 700 ms after that) using the EGI Net Station tool; (4) artifact detection by following the same rejection criteria as those stated in the processing of resting-state EEG data using the EGI Net Station tool; (5) baseline correction in Matlab; (6) averaging across segments (trials with Pz voltage exceeding 50 μV were excluded from the averaging [47]) for each condition (i.e., standard or target) and each participant in Matlab; (7) re-reference using the average reference (AVG) method in Matlab.

**Figure 2:**
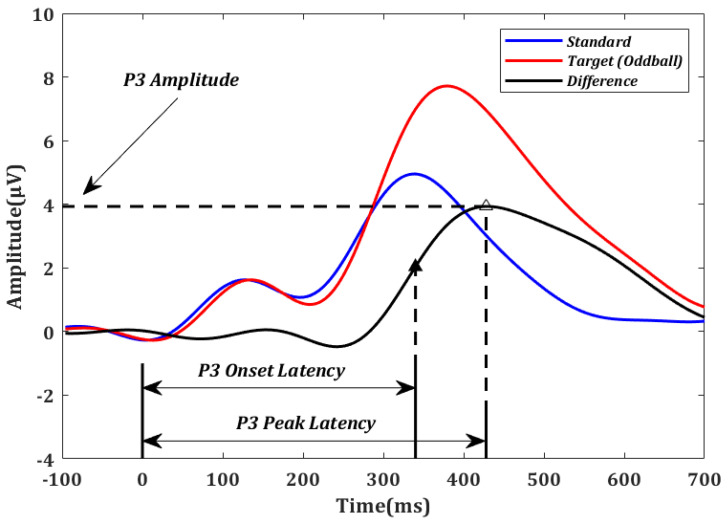
Event-related potentials (ERPs) averaged over all oddball (red) and standard (blue) trials across all participants in electrode Pz. For illustration purposes, the difference between the oddball and standard curves (black) was used to determine the P3 amplitude, P3 onset latency (solid triangle), and P3 peak latency (empty triangle) for each subject.

P3 amplitude and latency were estimated using the difference in ERP parameters between target and standard stimuli [48] at the Pz electrode. P3 amplitude was defined as the largest amplitude within [300 ms, 650 ms] [49], which is consistent with the ERPs averaged across all the participants. P3 onset latency was defined as the earliest time point at which an ERP amplitude exceeds half of the P3 peak amplitude when moving backward in time starting at the peak. The onset latency was demonstrated to be optimal under many conditions [50,51,52,53]. For comparison purposes, P3 peak latency was also calculated as the time point of the P3 peak amplitude. The method used to calculate P3 amplitude, P3 onset latency, and P3 peak latency is illustrated in Figure 2.

#### 2.3.4. Global Network Properties

With 90 × 90 coherence matrix W from the resting-state DASL image and 21 × 21 coherence matrix W from resting-state EEG data of each participant, the following global network properties were calculated according to previously defined network metrics [54]:

Clustering Coefficient (*CC*) is defined as the fraction of triangles around an individual network node as Equation (2) shows.

(2) $$CC=\frac{1}{N}\underset{i\in \theta }{\sum }\frac{\sum_{j,l\in \theta }{\left(w_{ij}w_{il}w_{jl}\right)}^{\frac{1}{3}}}{\sum_{j\in \theta }w_{ij}\left(\sum_{j\in \theta }w_{ij}-1\right)}$$

where *w_ij_* is the coherence value between region *i* and region *j*, θ is the set of all regions, and *N* is the total number of regions.

Characteristic path length (*CPL*) is defined as the mean value of the shortest path length between all pairs of network nodes as Equation (3) shows.

(3) $$CPL=\frac{1}{N}\underset{i\in \theta }{\sum }\frac{\sum_{j\in \theta ,i\neq j}d_{ij}}{N-1}$$

where *d_ij_* is the shortest path length between region *i* and region *j*, and the direct path length between region *i* and region *j* is 1/*w_ij_*.

Mean functional connectivity (*MFC*) is related to the mean value of all of the existing connections between each pair of nodes, which is defined in Equation (4).

(4) $$MFC=\frac{1}{N\times \left(N-1\right)}\underset{i,j\in \theta ,i\neq j}{\sum }w_{ij}$$

Global efficiency (*Ge*) is the average inverse shortest path length in the network. It is calculated as Equation (5).

(5) $$Ge=\frac{1}{N}\underset{i\in \theta }{\sum }\frac{\sum_{j\in \theta ,j\neq i}d_{ij}^{-1}}{N-1}$$

All four network properties, *MFC*, *Ge*, *CC*, and *L*, reflect the capability that brain resting-state networks transfer and process information globally.

#### 2.3.5. Correlation of P3 Properties with Task Performance and Global Network Properties

Correlation analysis was used to identify the association between the P3 properties and the global network properties, and between P3 properties and task performance (number of correct responses, reaction time) across participants. The reaction time is defined as the average time for correct responses. Due to potential outliers and violation of normal distribution from repeated data of the same participants, Shapiro–Wilk tests were used to detect whether the P3 properties, task performance, and global network properties were from normal distributions. For any variable that was rejected from the normality test, the Spearman rank correlation analysis was used instead of Pearson correlation analysis. A correlation with *p* < 0.05 is considered statistically significant. Family-wise error (FWE) corrections were performed to guard against false positives from multiple comparisons with corrected *p* < 0.05.

To assess the spatial distribution of global (mean) functional connectivity associated with P3 properties, the resting-state functional connectivity between each pair of nodes (*W_ij_*) was correlated with P3 properties across participants. The pairwise correlation type (either Spearman rank correlation or Pearson correlation) was the same as that adopted in the correlation analysis of mean functional connectivity and P3 properties. Only those edges (pairs of nodes) whose resting-state functional connectivity was correlated with P3 properties with uncorrected *p* < 0.001 were considered a significant connection. FWE corrections were performed to control false positive rates from multiple comparisons with corrected *p* < 0.05.

#### 2.3.6. Prediction of P3 Properties from rsFC Using rsDASL

To determine whether global rsFC (i.e., *MFC* from Equation (4)) can predict P3 properties in unseen individuals, a leave-one-out cross validation procedure was used. For each set of n − 1 participants, linear regression models were constructed to relate global rsFC to P3 properties, the built models were used to predict the P3 properties of the left-out participant using the global rsFC of this individual. We also compared the prediction accuracy using global rsFC with that using rsFC over an individual edge or using rsFC over multiple edges. For each set of n − 1 participants, linear regression models were constructed to relate rsFC over the most significant edge (an individual edge with the most significant correlation) to P3 properties, and over the most significant edges (multiple edges with the most significant correlations, defined as uncorrected *p* < 0.001) was selected from the training data set and used to predict the P3 properties of the left-out participant. Because of the aforementioned distributions, rank-based linear regression models were used for P3 amplitude, while regular linear regression models were used for P3 onset latency.

After prediction, correlation analysis between the observed and predicted P3 properties (Pearson correlation for P3 amplitude, Spearman correlation for P3 onset latency) was used to assess the prediction power. Steiger’s z and p were used to compare the correlation of prediction using global rsFC, rsFC over the most significant edges, and rsFC over the most significant edge.

## 3. Results

### 3.1. P3 Amplitude, P3 Latencies, Reaction Time, and Number of Correct Responses

P3 amplitude, P3 latencies, task reaction time, and number of correct responses varied across participants, with a mean P3 amplitude of 4.95 ± 2.14 μV, a mean P3 onset latency of 365.68 ± 63.53 ms, a mean P3 peak latency of 453.68 ± 51.63 ms, a mean task reaction time of 362.81 ± 23.45 ms, and a mean number of correct responses of 178.37 ± 28.72. As shown by Shapiro–Wilk tests, P3 amplitude and the number of correct responses were not normally distributed, while P3 latencies (onset latency and peak latency) and reaction time were normally distributed.

The correlation analysis indicated that there was a significant relationship between P3 amplitude and the P3 onset latency (r = −0.48, *p* = 0.039) and between P3 onset latency and the P3 peak latency (r = 0.80, *p* < 0.001), and a marginally significant relationship between P3 amplitude and the P3 peak latency (r = −0.42, *p* = 0.075).

### 3.2. Relations between P3 Properties and Task Performance

**Figure 3:**
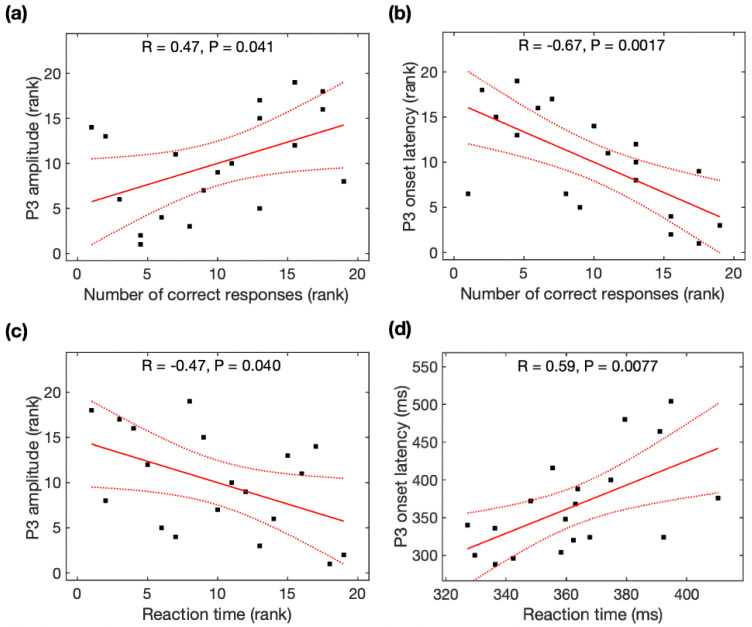
Relationship between the P3 parameters and task performance (number of correct responses and reaction time). The number of correct responses was correlated with (**a**) P3 amplitude and (**b**) P3 onset latency. Reaction time was correlated with (**c**) P3 amplitude and (**d**) P3 onset latency. Black squares are individual data points; solid red lines are linear regression lines; dotted red lines are 95% confidence intervals.

Spearman rank correlation analysis was used to identify the association between the number of correct responses and P3 amplitudes, between the number of correct responses and P3 latencies, and between the reaction time and P3 amplitudes because of non-normality for the number of correct responses and P3 amplitude across participants, while Pearson correlation analysis was used to identify the association between the reaction time and P3 latencies. The number of correct responses was significantly associated with P3 amplitudes (r = 0.47, *p* = 0.041, Figure 3a), P3 onset latencies (r = −0.67, *p* = 0.0017, Figure 3b), and P3 peak latencies (r = −0.51, *p* = 0.025, not shown). Reaction time was significantly associated with P3 amplitudes (r = -0.47, *p* = 0.040, Figure 3c) and P3 onset latencies (r = 0.59, *p* = 0.0077, Figure 3d), but marginally associated with P3 peak latencies (r = 0.43, *p* = 0.063). With the average of the data before and after meditation, task performance was marginally associated with P3 onset latencies but not associated with P3 amplitudes (see Appendix B).

### 3.3. Relations between P3 Properties and Global Network Properties Derived from Resting-State EEG

**Figure 4:**
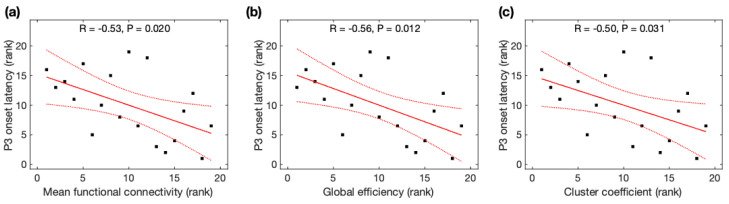
Relationship between P3 onset latency and global network properties derived from resting-state EEG. The rank of P3 onset latency was correlated with the rank of (**a**) mean function connectivity, (**b**) global efficiency, and (**c**) cluster coefficient. Black squares are individual data points; solid red lines are linear regression lines; dotted red lines are 95% confidence intervals.

Due to non-normality for the global efficiency, mean functional connectivity, cluster coefficient, and characteristic path length across participants, Spearman rank correlation analysis was used to identify their association with P3 properties. P3 onset latencies were significantly negatively correlated with *MFC* (r = −0.53, *p* = 0.020), *Ge* (r = −0.56, *p* = 0.012), and *CC* (r = −0.50, *p* = 0.031) (Figure 4), but not correlated with *CPL* (r = +0.35, *p* = 0.14). All correlations remained significant after family-wise error (FWE) correction. As observed with the average data before and after meditation, P3 onset latencies were significantly correlated with *MFC* and *Ge*, and marginally correlated with *CC*, but became insignificant after FWE correction (see Appendix B). However, P3 amplitudes were insignificantly correlated with all four network properties (*MFC*: r = +0.46, *p* = 0.049; *Ge*: r = +0.47, *p* = 0.041; *CC*: r = +0.45, *p* = 0.052; *CPL*: r = −0.52, *p* = 0.023) after FWE correction.

### 3.4. Relations between P3 Properties and Global Network Properties Derived from Resting-State DASL (rsDASL) fMRI

**Figure 5:**
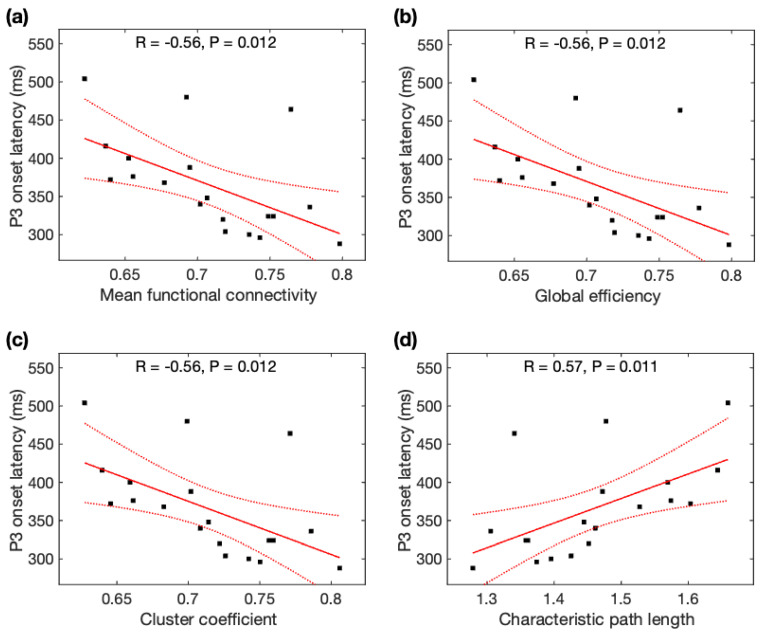
Relationship between P3 onset latency (ms) and global network properties derived from resting-state ASL fMRI: P3 onset latency was correlated with (**a**) mean function connectivity, (**b**) global efficiency, (**c**) cluster coefficient, and (**d**) characteristic path length. Black squares are individual data points; solid red lines are linear regression lines; dotted red lines are 95% confidence intervals.

Given the normal distributions of P3 onset latencies, P3 peak latencies, and rsDASL global network properties across participants, Pearson correlation analysis was used to identify its association with global network properties. P3 onset latencies were significantly negatively correlated with *MFC* (r = −0.56, *p* = 0.012), *Ge* (r = −0.56 *p* = 0.012), and *CC* (r = −0.56, *p* = 0.012), but positively correlated with *CPL* (r = +0.57, *p* = 0.011) (Figure 5). All correlations of P3 onset latencies remained significant after family-wise error (FWE) correction. As seen with the average data before and after meditation, P3 onset latencies were significantly correlated with *MFC*, *Ge*, *CC*, and *CPL*, and remained significant after FWE correction (see Appendix B). However, P3 peak latencies were not significantly correlated with any of the four network properties (*p* > 0.05).

**Figure 6:**
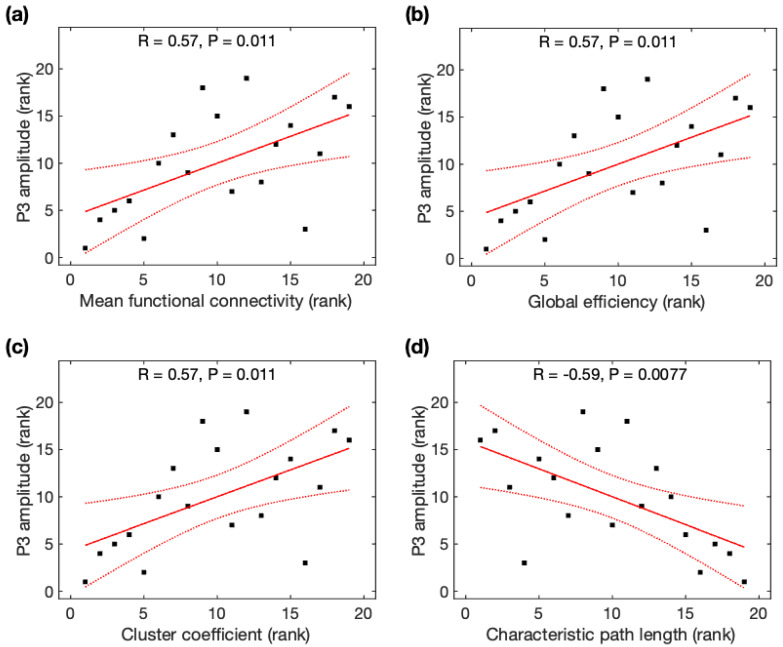
Relationship between P3 amplitude and global network properties derived from resting-state ASL fMRI. The rank of P3 amplitude was correlated with the rank of (**a**) mean function connectivity, (**b**) global efficiency, (**c**) cluster coefficient, and (**d**) characteristic path length. Black squares are individual data points; solid red lines are linear regression lines; dotted red lines are 95% confidence intervals.

Due to the non-normality of P3 amplitudes across participants, Spearman rank correlation analysis was used to identify its association with global network properties. P3 amplitudes were significantly positively correlated with *MFC* (r = 0.57, *p* = 0.011), *Ge* (r = 0.57, *p* = 0.011), and *CC* (r = 0.57, *p* = 0.011), but negatively correlated with *CPL* (r = −0.59, *p* = 0.0077) (Figure 6). All correlations remained significant after family-wise error (FWE) correction. Based on the average data before and after meditation, P3 amplitudes were significantly correlated with *MFC*, *Ge*, *CC*, and *CPL*, but became insignificant after FWE correction (see Appendix B). Using Steiger’s z tests, no significant difference was observed for the association of P3 amplitudes and global network properties using rsDASL and resting-state EEG (*p* > 0.05) despite an average increase of 21% in the four correlation coefficients with rsDASL.

### 3.5. Distribution of Edges Significantly Related to P3 Properties

**Figure 7:**
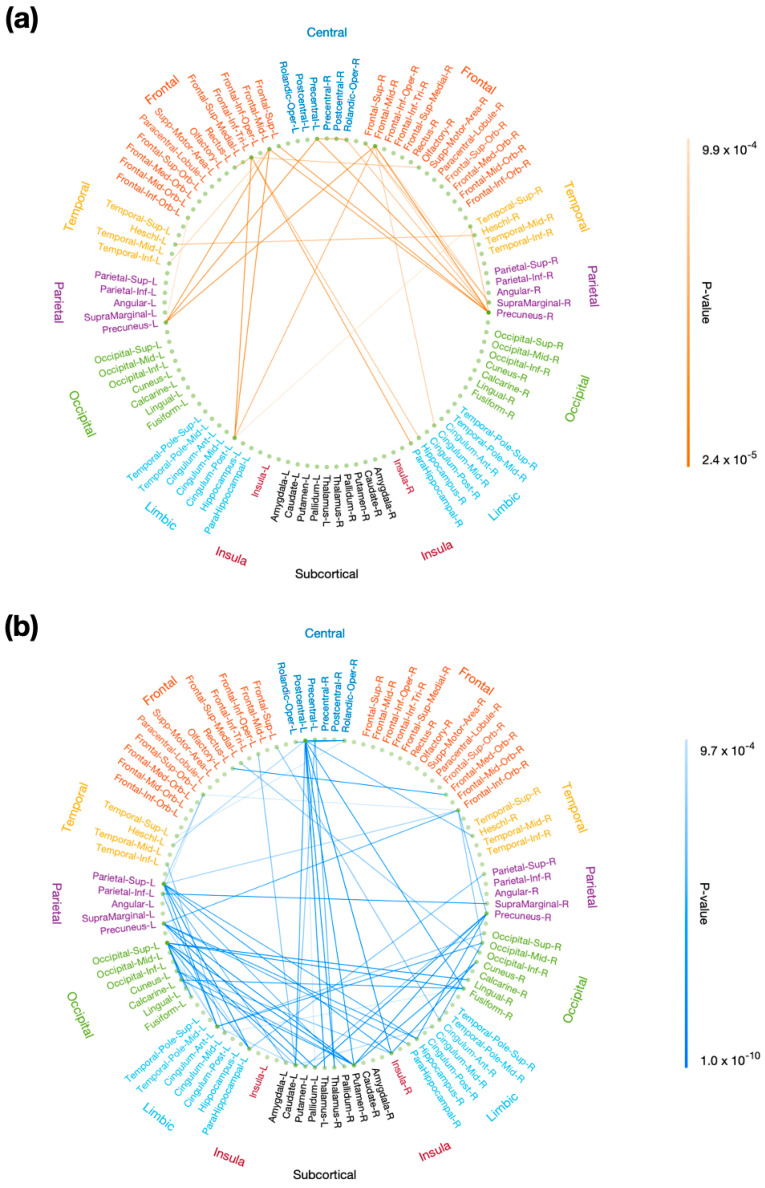
Correlation of resting-state functional connectivity with P3 properties. Resting-state functional connectivity, measured with rsDASL, was significantly correlated with (**a**) P3 onset latency (*p* < 0.001) and (**b**) P3 amplitude (*p* < 0.001). Blue edges represent positive correlation, while red edges represent negative correlation.

**Figure A1:**
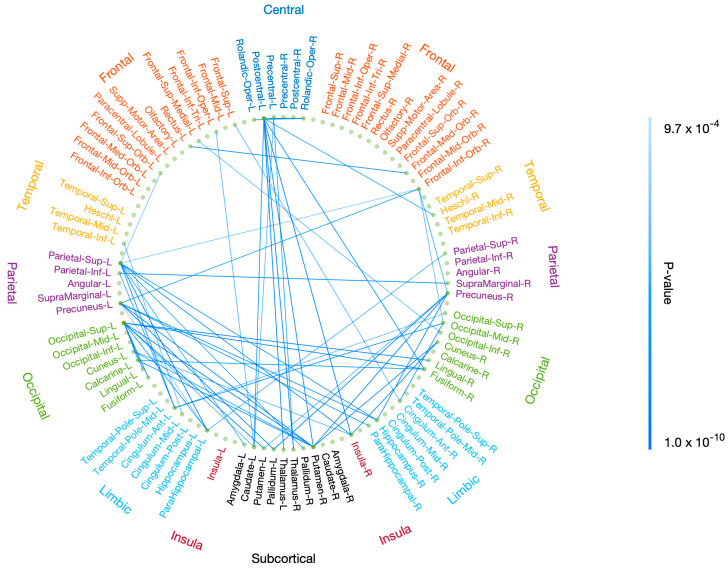
Correlation of resting-state functional connectivity with P3 properties after FWE correction. Resting-state functional connectivity, measured with rsDASL, was significantly correlated with P3 amplitude after FWE correction (*p* < 0.05). Blue edges represent positive correlation.

Using rsDASL, with uncorrected *p* < 0.001, the edges whose resting-state functional connectivity was significantly correlated with P3 onset latency were primarily those long-range connections between the prefrontal and parietal/limbic regions (Figure 7a), while the edges whose resting-state functional connectivity was significantly correlated with P3 amplitude were those between the prefrontal and parietal/occipital, between the central and subcortical, and between the limbic/subcortical and parietal/occipital regions (Figure 7b). However, after FWE correction, all edges lost significance for their correlation with P3 onset latency, while only 11 out of 68 edges lost significance for their correlation with P3 amplitude (Figure A1). These remaining edges showed functional connectivity in the aforementioned locations as in Figure 7b. Using resting-state EEG, no significant correlation with P3 properties was observed with uncorrected *p* < 0.001.

### 3.6. Prediction of P3 Properties from rsFC Using Resting-State EEG

Using resting-state EEG, observed P3 onset latency was marginally correlated with predicted P3 onset latency from the global *MFC* (r = +0.39, *p* = 0.097), but not correlated with the rsFC over the most significant edge (r = −0.080, *p* = 0.75). We found a marginally significant difference in the prediction power of P3 onset latency from the global rsFC and rsFC over the most significant edge (Steiger’s z = 1.37, *p* = 0.085).

Observed P3 amplitude was not significantly correlated with predicted P3 amplitude from the global *MFC* (r = +0.31, *p* = 0.19) or the rsFC over the most significant edge (r = +0.12, *p* = 0.61). We also found no difference in the predictive power of P3 amplitude from the global rsFC and rsFC over the most significant edge (Steiger’s z = 0.64, *p* = 0.26).

### 3.7. Prediction of P3 Properties from rsFC Using rsDASL

**Figure 8:**
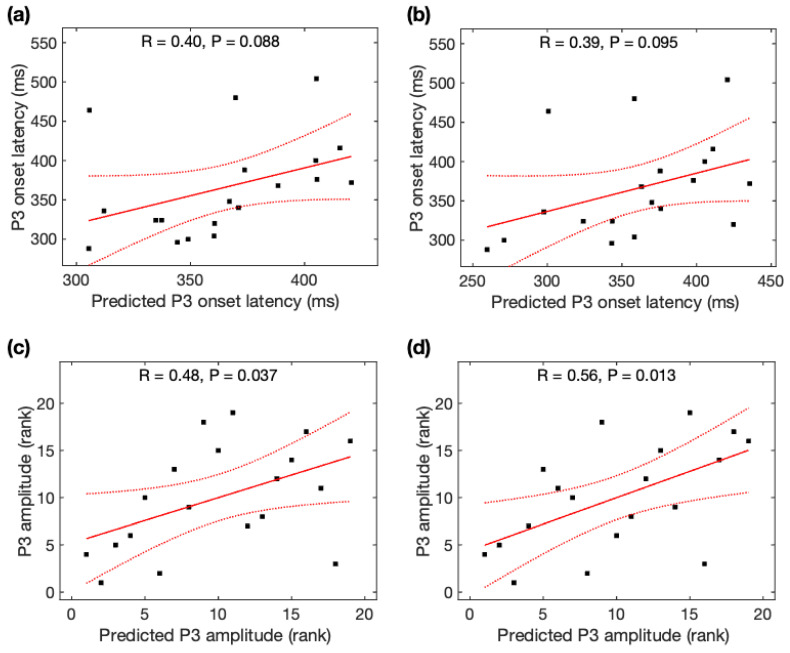
Relationship between predicted P3 properties and observed P3 properties. Observed P3 onset latency was marginally correlated with predicted P3 onset latency (**a**) from the global rsFC and (**b**) from the rsFC over the most significant edges. Observed P3 amplitude was significantly correlated with predicted P3 amplitude (**c**) from the global rsFC and (**d**) from the rsFC over the most significant edges. Black squares are individual data points; solid red lines are linear regression lines; dotted red lines are 95% confidence intervals.

Observed P3 onset latency was marginally correlated with predicted P3 onset latency from the global *MFC* (r = +0.40, *p* = 0.088) (Figure 8a) and from the rsFC over the most significant edges (r = +0.39, *p* = 0.095) (Figure 8b), but not correlated with the rsFC over the most significant edge (r = +0.11, *p* = 0.64). We found a marginally significant difference in the prediction power of P3 onset latency from the global rsFC and rsFC over the most significant edge (Steiger’s z = 1.33, *p* = 0.092).

Observed P3 amplitude was significantly correlated with predicted P3 amplitude from the global *MFC* (r = +0.48, *p* = 0.037) (Figure 8c) and from the rsFC over the most significant edges (r = +0.56, *p* = 0.013) (Figure 8d), but not correlated with the rsFC over the most significant edge (r = +0.12, *p* = 0.61). We found a marginally significant difference in the prediction power of P3 amplitude from the global rsFC and rsFC over the most significant edge (Steiger’s z = 1.52, *p* = 0.064). Using Steiger’s z tests, no significant difference was observed for the association of observed P3 amplitudes and predicted P3 amplitudes using rsDASL and resting-state EEG (*p* > 0.05) despite an increase of 55% in the *MFC* correlation coefficient with rsDASL.

## 4. Discussion

We have demonstrated that both P3 amplitude and P3 onset latency are significantly correlated with mean functional connectivity, global efficiency, cluster coefficient, and characteristic path length over the entire brain, using rsDASL. The global network metrics reflect interaction and integration of brain networks. P3 amplitude is directly proportional to the level of task-related attention, while P3 latency reflects the speed of information (stimulus) processing [11,55]. Our results suggest that a global brain interaction is related to the brain attention level, and that a globally efficient brain network may provide more efficient information processing. Our study also indicates that the neural mechanism of P3 amplitude and latency may be more closely related to brain global integration than connection between isolated regions.

The results are generally consistent with a recent investigation [36]; however, that study reported that the global resting-state metrics, measured by resting-state EEG, were only marginally related to P3 latency. The authors commented that the nonsignificant correlation with P3 latency was mainly caused by its inaccurate estimation. We found a significant correlation of P3 latency with global network metrics in our study. The detection of the significant correlation may be attributed to the additional data cleaning steps used in our study (e.g., removing eye movements by the global regression method) and the approach used to improve the accuracy of measuring P3 latency. In addition, we observed 21% larger correlation coefficients of P3 amplitude with global network metrics estimated from rsDASL compared to those from resting-state EEG (though these differences did not reach statistical significance). This result indicates that rsDASL can provide more robust estimates for global network metrics, which emerge from its minimal susceptibility to physiological noises and motion. Further studies are warranted to confirm this result with larger samples.

We found that both P3 amplitude and latency were correlated with the number of correct responses, indicating the link between the P3 parameters and behavioral task performance while performing an attention task. We also observed that the P3 parameters were related to global network efficiencies (especially those measured with rsDASL) at resting state. Therefore, these global network efficiencies, which can be measured conveniently with perfusion MRI while the participant is at rest, may serve as a neural index of attention. These results agree with a recently suggested neuromarker of attention from whole-brain functional connectivity [56]. Although they used the connections that were significantly related to gradCPT attention task, the connections were chosen from pairwise connections across the entire brain, supporting the understanding that the human attention ability involves coordination of many brain networks. In addition, using rsDASL, global rsFC over the entire brain has shown marginally significant prediction power of P3 properties compared to rsFC over an individual edge for unseen individuals. The observed P3 amplitudes had 55% larger correlation with predicted P3 amplitudes from rsDASL than those from resting-state EEG, although no statistical significance was found. The lack of statistical difference is likely caused by our small sample size. This result suggests that the whole-brain intrinsic connectivity—resting-state functional connectivity using rsDASL—may serve as an index of neural processes underlying components of attention indexed by the well-known P3. The measure of attention, only requiring the use of resting-state data, can be especially helpful for the populations who have difficulty in performing tasks (such as ADHD). A large independent dataset is warranted to validate the measure.

The brain connections that were significantly correlated with P3 onset latency were mainly those long-range connections between the prefrontal and parietal/limbic regions. In contrast, the significant connections with P3 amplitude were those between the prefrontal and parietal/occipital, between the central and subcortical, and between the limbic/subcortical and parietal/occipital regions. These results emphasize that the P3 attentional processes do not only rely on brain connections in traditional attention regions—prefrontal and parietal/occipital regions [48,57,58,59]—but also incorporate information from subcortical [60] and limbic [61,62,63] regions. Our results support prior research that indices of attention emerge from coordinated activities across the entire brain [8,56]. The circuits connecting frontal and parietal cortices have been shown to play a crucial role in top-down/bottom-up attention control [64] and serve as a direct corticocortical pathway in work memory tasks [65,66]. Working memory tasks were also reported as involving an indirect pathway between frontal and parietal regions via subcortical regions (basal ganglia and thalamus) [65]. Our findings for the significant correlation of P3 amplitude with parietal-subcortical rsFC support the indirect frontal and parietal pathway via subcortical regions. In contrast, no significant correlation of P3 amplitude with frontal-subcortical rsFC was observed, which may have been due to the low difficulty level of our oddball task without need of interaction with frontal regions. Parietal-limbic (e.g., hippocampal regions) rsFC was correlated with P3 amplitude, agreeing well with the P3 generation from hippocampal areas using implanted intracranial electrodes [61,63]. The correlation of frontal-occipital rsFC with P3 amplitude is consistent with previous studies [36,38]. This finding suggests that stronger connection with occipital region enables more attentional resources to visual stimulus and so improved target detection during the visual oddball task. rsFC between central (precentral/postcentral) and subcortical (basal ganglia and thalamus) regions was correlated with P3 amplitude. The precentral and postcentral regions are sensory and motor areas, while basal ganglia and thalamus regions are responsible for sensory gating of motor control [67] and relaying information from sensory and motor regions [68], respectively. This result supports the evidence that basal ganglia and thalamus are engaged in evaluating and responding to the target stimulus [69].

This study has limitations. First, it has a limited sample size. To increase the statistical power, we have included data both before and after meditation training as independent data. However, we have verified that the correlations of data before and after meditation training are insignificant. In addition, we used independent samples by averaging the data before and after meditation training to confirm the trend of these results. Second, the participants were college students from a meditation course, serving as a convenient sample. Therefore, our results may not be generalized to a healthy population at large because the sample was not randomly selected. Third, direct comparison of rsDASL with resting-state BOLD fMRI was not performed. Resting-state BOLD fMRI remains a popular method for the analyses of brain functional connectivity. Therefore, it is worth investigating whether global network properties derived from BOLD fMRI can be an alternative to characterize P3-related attention. Fourth, the extension of the global network properties to clinical population (e.g., ADHD) requires further investigation of their correlation with clinical measures of attention deficits (such as the ADHD rating scale [70]). Future studies with large, independent, and random samples are required to confirm the association between P3 parameters and brain global network properties and between clinical measures of attention deficits and brain global network properties.

## 5. Conclusions

Our significant findings using rsDASL support further investigation into the power of the technique and P3 correlation of brain global functional connectivity. Our results show that indices of attention emerge from coordinated activities across the entire brain. We also offer a single global index, which can characterize neural processes underlying components of attention indexed by the well-known P3. The global index may serve as a neuromarker of attention for studies investigating neuropsychiatric disorders involving attentional impairments (e.g., ADHD) if confirmed in a larger study.

## Data Availability

Raw data were generated from MRI scanner and EEG. MRI reconstruction software is the vendor’s proprietary product. The sharing of derived data will be supported by direct request. After publishing our main findings, requests for data will be evaluated on a case-by-case basis. Before sharing data, we will make sure that all data are free of identifiers that could directly or indirectly link information to an individual and that all sharing is compliant with institutional and IRB policies.

## Appendix A

We calculated Pearson correlation coefficients of global network properties before and after meditation training and correlation coefficients of p3 parameters before and after meditation training.

We did not find significant correlations before and after meditation training after family-wise error (FWE) correction using rsDASL (*MFC*: *p* = 0.14; *CC*: *p* = 0.14; *GE*: *p* = 0.14; *CPL*: *p* = 0.092), using EEG (*MFC*: *p* = 0.19; *CC*: *p* = 0.16; *GE*: *p* = 0.24; *CPL*: *p* = 0.17), and using ERP (P3 amplitude: *p* = 0.036; P3 onset latency: *p* = 0.19; P3 peak latency: *p* = 0.11).

## Appendix B

### Appendix B.1. Relations between P3 Properties and Task Performance

With the average data before and after meditation, the number of correct responses was not significantly associated with P3 amplitudes (r = 0.39, *p* = 0.26) but marginally associated with P3 onset latencies (r = −0.63, *p* = 0.052). Reaction time was not significantly associated with P3 amplitudes (r = −0.45, *p* = 0.20) but marginally associated with P3 onset latencies (r = 0.62, *p* = 0.058).

### Appendix B.2. Relations between P3 Properties and Global Network Properties Derived from Resting-State EEG

As observed with the average data before and after meditation, P3 onset latencies were significantly correlated with *MFC* (r = −0.64, *p* = 0.047) and *Ge* (r = −0.67, *p* = 0.033), and marginally correlated with *CC* (r = −0.59, *p* = 0.072), but became insignificant after FWE correction.

### Appendix B.3. Relations between P3 Properties and Global Network Properties Derived from Resting-State DASL (rsDASL) fMRI

As seen with the average data before and after meditation, P3 onset latencies were significantly correlated with *MFC* (r = −0.79, *p* = 0.0065), *Ge* (r = −0.79, *p* = 0.0065), *CC* (r = −0.79, *p* = 0.0065), and *CPL* (r = 0.79, *p* = 0.0067), and remained significant after FWE correction; P3 amplitudes were significantly correlated with *MFC* (r = 0.69, *p* = 0.027), *Ge* (r = 0.69, *p* = 0.027), *CC* (r = 0.69, *p* = 0.027), and *CPL* (r = −0.71, *p* = 0.020), but became insignificant after FWE correction.
